# Tamoxifen therapy benefit in luminal A and B breast cancer with 20-year follow-up: Secondary analysis: Stockholm Tamoxifen-2, 3, and 5 randomized controlled trials

**DOI:** 10.1093/jnci/djag049

**Published:** 2026-02-19

**Authors:** Oscar Danielsson, Huma Dar, Anna Nordenskjöld, Gizeh Perez-Tenorio, Bo Nordenskjöld, Tommy Fornander, Olle Stål, Nicholas P Tobin, Julia Tutzauer, Annelie Johansson, Linda S Lindström

**Affiliations:** Department of Oncology and Pathology, Karolinska Institutet and University Hospital, Stockholm, Sweden; Breast Center, Karolinska Comprehensive Cancer Center, Karolinska University Hospital, Stockholm, Sweden; Department of Oncology and Pathology, Karolinska Institutet and University Hospital, Stockholm, Sweden; Breast Center, Karolinska Comprehensive Cancer Center, Karolinska University Hospital, Stockholm, Sweden; Department of Oncology, Institute of Clinical Sciences, Sahlgrenska Academy, Gothenburg, Sweden; Department of Medicine, Southern Älvsborgs Hospital, Borås, Sweden; Department of Biomedical and Clinical Sciences and Department of Oncology, Linköping University, Linköping, Sweden; Department of Biomedical and Clinical Sciences and Department of Oncology, Linköping University, Linköping, Sweden; Department of Oncology and Pathology, Karolinska Institutet and University Hospital, Stockholm, Sweden; Breast Center, Karolinska Comprehensive Cancer Center, Karolinska University Hospital, Stockholm, Sweden; Department of Biomedical and Clinical Sciences and Department of Oncology, Linköping University, Linköping, Sweden; Department of Oncology and Pathology, Karolinska Institutet and University Hospital, Stockholm, Sweden; Breast Center, Karolinska Comprehensive Cancer Center, Karolinska University Hospital, Stockholm, Sweden; Department of Oncology and Pathology, Karolinska Institutet and University Hospital, Stockholm, Sweden; Breast Center, Karolinska Comprehensive Cancer Center, Karolinska University Hospital, Stockholm, Sweden; Department of Oncology and Pathology, Karolinska Institutet and University Hospital, Stockholm, Sweden; Breast Center, Karolinska Comprehensive Cancer Center, Karolinska University Hospital, Stockholm, Sweden; Department of Oncology and Pathology, Karolinska Institutet and University Hospital, Stockholm, Sweden; Breast Center, Karolinska Comprehensive Cancer Center, Karolinska University Hospital, Stockholm, Sweden

## Abstract

**Background:**

Patients with estrogen receptor–positive breast cancer have a substantial late risk of distant recurrence, but the long-term subtype-specific tamoxifen benefit remains poorly understood.

**Methods:**

A secondary analysis of the Stockholm Tamoxifen randomized trials (1976-1997, *n* = 3930) with 20-year follow-up was conducted. Formalin-fixed, paraffin-embedded blocks were available for 2250 patients. A total of 952 patients with estrogen receptor–positive and HER2-negative tumors, classified as luminal A (*n* = 688) and luminal B (*n* = 264) using Agilent microarrays, were analyzed. Patients were randomly assigned to at least 2 years of tamoxifen therapy or no endocrine therapy. Distant recurrence-free interval was assessed by Kaplan–Meier analysis and multivariable Cox proportional hazards regression.

**Results:**

Patients with luminal A tumors had low early risk and modest early benefit from tamoxifen therapy (5-year distant recurrence-free interval treated vs control: 93% vs 89%; absolute difference = 4%) that increased over the 20-year follow-up (20-year distant recurrence-free interval: 76% vs 66%; absolute difference = 10%), highlighting long-term benefit. In contrast, patients with luminal B tumors had larger early risk and treatment benefit (5-year distant recurrence-free interval: 72% vs 57%; absolute difference = 15%), which remained stable over time (20-year distant recurrence-free interval: 55% vs 37%; absolute difference = 18%). Multivariable analyses showed that luminal patients benefited from tamoxifen therapy (luminal A: adjusted hazard ratio [HR] = 0.57, 95% confidence interval [CI] = 0.42 to 0.78; luminal B: adjusted HR = 0.68, 95% CI = 0.46 to 0.99). Patients with favorable tumor characteristics benefited regardless of luminal subtype.

**Conclusions:**

Tamoxifen reduces the long-term risk of distant recurrence in luminal A and B tumors, although timing of benefit varies by subtype. Even after treatment, patients with luminal tumors have a substantial late risk, highlighting the need for long-term follow-up.

**Clinical Trial Registration:**

The trial center for the Stockholm Tamoxifen trials was the Regional Cancer Center Stockholm-Gotland, in Stockholm, Sweden. The start of the Stockholm Tamoxifen trials in 1976 was before the custom of trial registration started in Sweden, therefore no trial number is available.

## Introduction

Patients with estrogen receptor–positive breast cancer have a substantial long-term risk of distant recurrence, even after several decades, with approximately half of all events occurring more than 5 years after diagnosis.^1-6^ Adjuvant endocrine therapy, commonly tamoxifen, is the standard treatment for estrogen receptor–positive breast cancer and is administered to virtually all patients with estrogen receptor–positive tumors.^7^ In addition to estrogen receptor positivity, treatment decisions are based on the patient’s clinical risk during the first 5-10 years after diagnosis, as estimated from the clinically used tumor characteristics, including tumor size, histological grade, lymph node status, and progesterone receptor status.^8-11^

Breast cancer is a heterogeneous disease, and molecular subtyping has identified at least 5 distinct subtypes that inform treatment strategies in clinical practice.^6^^,^^12-15^ Luminal A and luminal B subtypes are the most common in estrogen receptor–positive breast cancer, together constituting approximately 80% of all tumors. Although patients with luminal B tumors have a higher early risk of recurrence, long-term follow-up is needed to assess lifetime risk given the sustained long-term risk in patients with estrogen receptor–positive disease, especially for patients with initially less aggressive tumor characteristics. Therefore, it is essential to understand the long-term benefit from tamoxifen in these patients and how the clinically used tumor and patient characteristics influence benefit.

The lack of well-annotated clinical trials with complete long-term follow-up has resulted in the current clinical challenge to predict risk and endocrine therapy benefit.^16^ Long follow-up is essential for patients with estrogen receptor–positive breast cancer because of the long-term risk of distant recurrence. We used the Stockholm Tamoxifen (STO) randomized controlled trials, including premenopausal and postmenopausal patients with complete 20-year high-quality follow-up. In this study, we aimed to investigate the long-term benefit of tamoxifen therapy in estrogen receptor–positive and HER2-negative breast cancer patients with luminal A or B subtype. In addition, we investigated the impact of the clinically used tumor characteristics and genomic risk on tamoxifen benefit within each luminal subtype.

## Methods

### The STO trials

**Figure 1.**
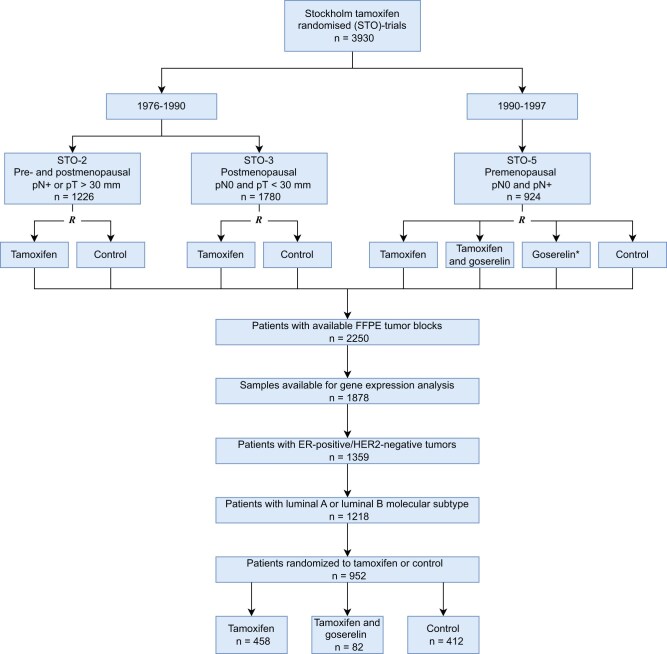
Consort diagram of the Stockholm Tamoxifen (STO) 2, 3, and 5 randomized controlled trials. Molecular subtype using the PAM50 gene expression classification. Abbreviations: ER = estrogen receptor; FFPE = formalin-fixed paraffin-embedded; pN = number of positive lymph nodes; pT = primary tumor. *Patients randomly assigned to goserelin only were excluded.

The STO trials enrolled patients with invasive breast cancer between 1976 and 1997, and premenopausal and postmenopausal patients at low and high clinical risk were included.^17-21^ In total the trials included 3930 patients; formalin-fixed, paraffin-embedded tumor blocks were available for 2250 patients. Of the patients with available formalin-fixed, paraffin-embedded blocks, 1878 had annotated Agilent gene-expression profiling, and 1359 patients had estrogen receptor–positive and HER2-negative tumors. Of these patients, 1218 had tumors that were classified as luminal A or B subtype, and 952 of these patients were randomly assigned to tamoxifen or control, excluding patients who only received goserelin or were not randomly assigned to endocrine treatment (see Figure 1). At inclusion, patients were stratified by lymph node status and tumor size. All patients included in the trials were clinically free of distant recurrence at primary diagnosis and underwent either full mastectomy or breast-conserving surgery with additional postoperative local breast radiotherapy. Following surgery, postmenopausal patients in the STO-2 and STO-3 trials were randomly assigned to receive at least 2 years of tamoxifen therapy (40 mg daily) vs no tamoxifen therapy (control). Premenopausal patients in the STO-5 trial were randomly assigned to 2 years endocrine therapy (tamoxifen, goserelin, combination) or control. Patients who were randomly assigned to goserelin only were excluded from this study because of the demonstrated differential benefit as compared with tamoxifen therapy in a previous study.^22^ For more details, see Supplementary Methods.

Follow-up until December 31, 2016, was obtained from Swedish national and regional registries of high validity and essentially complete coverage.^23-25^ Detailed patient and clinical information was available for all patients in the trials. The STO trials were approved by the Karolinska Institutet regional ethics committee with the Stockholm Regional Cancer Center as the trial center. Informed consent was obtained before random assignment. The trials were approved and initiated before the practice of trial registration in Sweden.

### Immunohistochemistry

Immunohistochemical analysis for estrogen receptor, progesterone receptor, HER2, and Ki-67 was performed in 2014 (STO-3) and 2020 (STO-2 and STO-5). The percentage of cancer cells positive for estrogen receptor, progesterone receptor, HER2, and Ki-67 was scored by experienced breast cancer pathologists. Estrogen receptor–positive and progesterone receptor–positive status were defined by a threshold of 10% or greater according to Swedish National guidelines,^7^ and Ki-67 was measured in the whole tumor section and categorized as low (<15%) and intermediate/high (≥15%). HER2 positivity was defined as intensity at least 3 by immunohistochemical analysis.

### Tumor size and histological tumor grade

Tumor diameter was measured according to clinical guidelines, and tumor grade was assessed according to the Nottingham Histological Score system (Elston grade).^26^

### Genomic risk

Primary tumors were classified as having genomic high risk or low risk based on Agilent microarray gene expression profiling using the 70-gene signature (MammaPrint).^27^

### Molecular subtypes (PAM50)

Agilent microarray gene expression profiling data on primary tumors were used to assign each tumor to 1 of 5 molecular subtypes (luminal A, luminal B, HER2-enriched, basal-like, normal-like) using the PAM50 gene expression classification, as described by Parker et al.^28^ For more details, see Supplementary Methods.

### Statistical analyses

Patients with estrogen receptor–positive and HER2-negative breast cancer with luminal A and B subtype tumors were included in the analyses (see Figure 1). The clinical endpoint for all analyses was distant recurrence-free interval with distant recurrence (ie, distant metastatic disease) as the event.^29^ In patients with missing date of distant metastasis, breast cancer death was used (*n* = 9). Follow-up started at the date of primary breast cancer diagnosis and ended at the date of distant recurrence, date of death, emigration from Sweden (11 women emigrated), or end of study follow-up (December 31, 2016).

The 20-year tamoxifen therapy benefit by luminal A and luminal B subtypes and the clinically used tumor characteristics was assessed using univariate Kaplan–Meier analysis and multivariable Cox proportional hazards regression. Statistical significance for Kaplan–Meier analysis was assessed using the log-rank test. Crude multivariable analyses were adjusted for age, year of breast cancer diagnosis, lymph node status, radiotherapy and menopausal status, which together defined trial stratification and treatment. Adjusted multivariable analyses included age, year of primary breast cancer diagnosis, tumor size, tumor grade, lymph node status, progesterone receptor status, Ki-67 status, type of surgery, chemotherapy, radiotherapy, and menopausal status. Patients with missing tumor characteristics (*n* = 47) were excluded from the adjusted multivariable analyses. In the stratified multivariable analyses, categorical covariates were adjusted only if each level had at least 10 patients.

Patient and tumor characteristics were divided into favorable vs unfavorable groups (ie, small or large tumor size, tumor grade 1-2 or 3, Ki-67 low or medium to high, lymph node-negative or lymph node-positive, progesterone receptor–positive or progesterone receptor–negative, premenopausal or postmenopausal status, and low or high genomic risk).

Sensitivity analysis excluding patients who received tamoxifen and goserelin (*n* = 82) yielded similar results.

All analyses were 2-sided, and a *P* value less than .05 was considered statistically significant. Data preparation and survival analyses were performed using R version 4.3.2.

## Results

The study included 952 estrogen receptor–positive and HER2-negative breast cancer patients with luminal A or luminal B tumors from the STO trials (Figure 1). Of the 952 patients in the cohort, 458 (48%) received tamoxifen therapy alone, 82 (9%) received tamoxifen plus goserelin, and 412 (43%) received no endocrine therapy (control). Patients receiving tamoxifen alone included 79 (8%) premenopausal patients and 379 (40%) postmenopausal patients. Patients receiving tamoxifen plus goserelin included 82 (9%) premenopausal patients and 0 (0%) postmenopausal patients. The control group included 82 (9%) premenopausal patients and 330 (35%) postmenopausal patients. Tumors were classified as luminal A in 688 (72%) patients and luminal B in 264 (28%) patients. Tumors with luminal B subtype were generally associated with more aggressive tumor characteristics compared with luminal A tumors (Table 1). Specifically, luminal B subtype was associated with lymph node positivity (lymph node–positive: luminal A: 24%; luminal B: 62%; *P* < .001) and higher tumor proliferation (Ki-67 high: luminal A: 13%; luminal B: 49%; *P* < .001). Additionally, genomic high risk, as assessed by the 70-gene signature, was seen for 62% of luminal B tumors compared with 19% of luminal A tumors (*P* < .001).

**Table 1.** Primary patient and tumor characteristics of ER-positive/HER2-negative breast cancer patients in the STO-trials

Primary tumor characteristics	Luminal A patients, No. (%) (*n* = 688)	Luminal B patients, No. (%) (*n* = 264)	*P* ^a^
Tumor size			
pT ≤ 20 mm	527 (77.3)	135 (51.5)	<.001
pT > 20 mm	155 (22.7)	127 (48.5)	
Unknown	6 (—)	2 (—)	
Tumor grade			
1	161 (23.6)	11 (4.2)	<.001
2	467 (68.5)	145 (55.6)	
3	54 (7.9)	105 (40.2)	
Unknown	6 (—)	3 (—)	
Lymph node status			
Negative	521 (75.7)	100 (37.9)	<.001
Positive	167 (24.3)	164 (62.1)	
Progesterone receptor status			
Positive	549 (80.5)	186 (70.7)	.0016
Negative	133 (19.5)	77 (29.3)	
Unknown	6 (—)	1 (—)	
Ki-67 status			
Low	577 (86.6)	133 (51.2)	<.001
High	89 (13.4)	127 (48.8)	
Unknown	22 (—)	4 (—)	
Genomic risk			
Low	560 (81.4)	100 (37.9)	<.001
High	128 (18.6)	164 (62.1)	
Menopausal status			
Pre	164 (23.8)	79 (29.9)	.057
Post	524 (76.2)	185 (70.1)	

a Fisher exact test was used to compute *P* values. Genomic risk was assessed using the 70-gene signature.

### Long-term tamoxifen therapy benefit by luminal A and B subtypes

**Figure 2.**
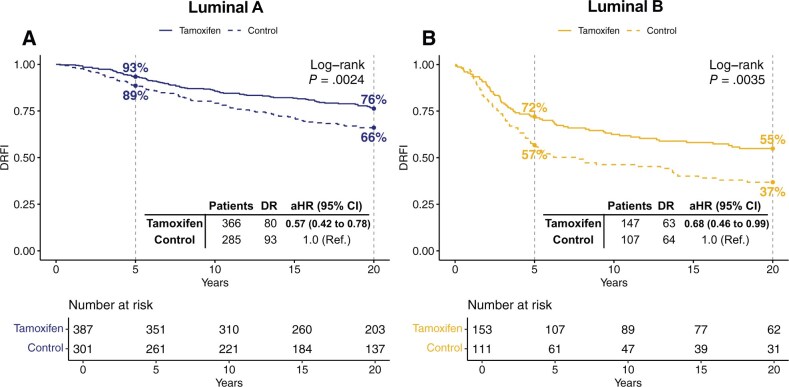
Kaplan–Meier and multivariable Cox proportional hazards regression analysis by molecular subtype. Kaplan–Meier analysis of 20-year distant recurrence-free interval (DRFI) in patients with estrogen receptor–positive/HER2-negative breast cancer by: **(A)** luminal A and **(B)** luminal B. Hazard ratios (aHR) were adjusted for age, year of primary breast cancer diagnosis, tumor size, tumor grade, lymph node status, progesterone receptor status, Ki-67 status, type of surgery, chemotherapy, radiotherapy, and menopausal status. Abbreviations: CI = confidence interval; DR = distant recurrence; DRFI = distant recurrence-free interval; Ref. = referent.

Univariate Kaplan–Meier analyses suggested a statistically significant long-term tamoxifen benefit for both luminal subtypes (luminal A, log-rank: *P* = .002; luminal B, log-rank: *P* = .004; Figure 2, A and B). For patients with luminal A subtype treated with tamoxifen therapy, the distant recurrence-free interval was 93% (95% confidence interval [CI] = 91% to 96%) at 5 years and 76% (95% CI = 72% to 81%) at 20 years compared with 89% (95% CI = 85% to 92%) and 66% (95% CI = 61% to 72%) in the endocrine untreated control arm (Figure 2, A). Further, for luminal A patients, the absolute difference in distant recurrence-free interval between the endocrine treated arm and the control arm was 4% at 5 years and 10% at 20 years, highlighting a long-term benefit from tamoxifen that increased over time. For patients with luminal B subtype, the distant recurrence-free interval was 72% (95% CI = 65% to 80%) at 5 years and 55% (95% CI = 47% to 64%) at 20 years for tamoxifen-treated patients vs 57% (95% CI = 48% to 67%) and 37% (95% CI = 29% to 47%) in the control arm (Figure 2, B). The absolute benefit was 15% at 5 years and 18% at 20 years, showing a substantial early benefit that persisted over the follow-up.

Multivariable Cox proportional hazards regression yielded similar results, with a statistically significant long-term benefit from tamoxifen for patients with luminal A (adjusted hazard ratio [HR] = 0.57, 95% CI = 0.42 to 0.78) and luminal B (adjusted HR = 0.68, 95% CI = 0.46 to 0.99) tumors (Figure 2, A and B). Similar results were observed using crude multivariable Cox proportional hazards regression (see the Table S1).

### Subgroup analyses by tumor and patient characteristics

**Figure 3.**
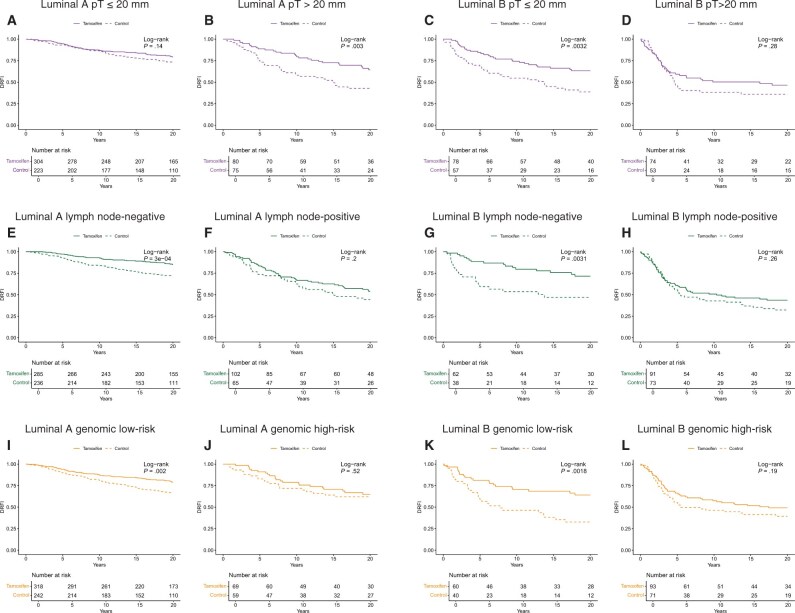
Kaplan–Meier by luminal subtype and tamoxifen treatment. Kaplan–Meier analysis of 20-year distant recurrence-free interval (DRFI) in patients with estrogen receptor–positive/HER2-negative breast cancer by luminal A and B subtype and tamoxifen therapy (tamoxifen, tamoxifen and goserelin, or control). Genomic risk was assessed using the 70-gene signature.

Subgroup analyses were performed to determine how tamoxifen benefit was influenced by the clinically used patient and tumor characteristics. Kaplan–Meier analyses were used to analyze distant recurrence-free interval by tumor size, Ki-67, tumor grade, progesterone receptor status, lymph node status, menopausal status, genomic risk, and luminal A and B subtypes (Figure 3; Figure S1).

**Figure 4.**
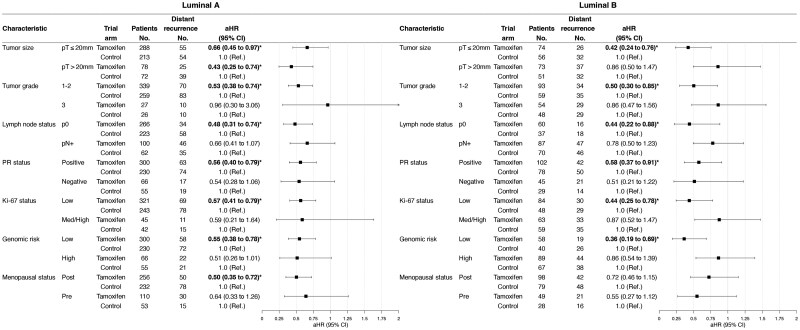
Multivariable Cox proportional hazards analysis of long-term tamoxifen therapy benefit by the clinically used tumor characteristics. Multivariable Cox proportional hazards regression analysis of distant recurrence-free interval by luminal subtype in patients with estrogen receptor–positive/HER2-negative breast cancer with 20 years of follow-up. Analyses were further stratified by tumor size, tumor grade, lymph node status, progesterone receptor (PR) status, Ki-67 status, genomic risk, and menopausal status. Models were adjusted for age, year of primary breast cancer diagnosis, tumor size, tumor grade, lymph node status, PR status, Ki-67 status, chemotherapy, radiotherapy, type of surgery, and menopausal status. Genomic risk was assessed using the 70-gene signature. Asterisk indicates statistical significance. Abbreviations: CI = confidence interval; aHR = adjusted hazard ratio; med = medium; pN = number of positive lymph nodes; pT = primary tumor; Ref. = referent.

In the Kaplan–Meier analysis, patients with favorable tumor characteristics had a substantial benefit from tamoxifen across both subtypes (Figure 3; Figure S1). Patients with luminal A tumors more consistently benefited from tamoxifen treatment, including patients with unfavorable characteristics, such as larger tumor size (primary tumor [pT] > 20 mm). For patients with luminal B tumors, a statistically significant benefit was seen with favorable tumor characteristics, whereas benefit attenuated in tumors with unfavorable characteristics (Figure 4). The same pattern was not seen for patients with luminal A tumors.

Notably, for patients at low clinical risk, for example patients with luminal A tumors of smaller tumor size (pT ≤ 20 mm), lymph node–negative tumors, or low genomic risk, the early risk of distant recurrence was low. However, for patients at low early risk, a substantial long-term risk of distant recurrence was observed, and the benefit from tamoxifen was seen mainly after 10-15 years. This underscores the importance of long-term follow-up especially for patients at lower clinical risk (Figure 3).

Multivariable analyses for patients with luminal A tumors showed that tamoxifen was associated with a statistically significant reduction in the long-term risk of distant recurrence in the favorable tumor characteristics groups. For instance, treated lymph node–negative patients with luminal A disease had a 52% reduction in long-term risk compared with the control group (adjusted HR = 0.48, 95% CI = 0.31 to 0.74), but a statistically significant benefit was not seen for lymph node–positive patients (Figure 4). Further, treated patients with luminal A genomic low-risk tumors had a statistically significant reduction in distant recurrence compared with endocrine untreated patients (adjusted HR = 0.55, 95% CI = 0.38 to 0.78), but a statistically significant benefit was not seen for patients with genomic high-risk tumors although the point estimates were similar. Similar findings were seen for Ki-67 and tumor grade, but numbers of patients were limited for tumor grade 3 and medium to high Ki-67 (see Figure 4). Finally, postmenopausal patients with luminal A tumors had a statistically significant benefit (adjusted HR = 0.50, 95% CI = 0.35 to 0.72), but this was not seen for premenopausal patients (Figure 4). Crude analyses yielded similar results (Figure S2).

Multivariable analyses for patients with luminal B tumors showed that tamoxifen was associated with a statistically significant reduction in the risk of distant recurrence in subgroups with favorable tumor characteristics (Figure 4). For instance, patients with smaller tumors (pT ≤ 20 mm) had a 58% reduced risk of distant recurrence (adjusted HR = 0.42, 95% CI = 0.24 to 0.76), lymph node–negative patients had a 56% reduced risk (adjusted HR = 0.44, 95% CI = 0.22 to 0.88), and patients with low genomic risk had a 64% reduced risk (adjusted HR = 0.36, 95% CI = 0.19 to 0.69) compared with the control group (Figure 4). Conversely, for patients with luminal B tumors with unfavorable tumor characteristics, a statistically significant benefit from tamoxifen was not seen (Figure 4). Crude analyses were consistent with the multivariable analyses (Figure S2).

## Discussion

This secondary analysis of the STO randomized trials with 20 years of follow-up demonstrates improvements in distant recurrence-free interval from tamoxifen therapy for patients with luminal A and luminal B tumor subtypes. However, the timing of benefit differed by subtype. An early sustained benefit was seen in patients with luminal B tumors, whereas in patients with luminal A tumors the early benefit was modest but increased over time. Despite tamoxifen therapy, patients had a substantial risk of distant recurrence that persisted well beyond 10 years after diagnosis, underscoring the need for long-term follow-up to assess late distant recurrence risk and tamoxifen benefit.

The importance of tamoxifen therapy for patients with estrogen receptor–positive disease is well established; however, little is known about the long-term, subtype-specific benefit. Most studies, including the hallmark meta-analysis by the Early Breast Cancer Trialists’ Collaborative Group,^16^ do not include molecular subtype information, and few studies have 20 years of follow-up. Long-term analyses have shown that the risk of distant recurrence remains high from 5 to 20 years after diagnosis, even after completing 5 years of adjuvant endocrine therapy, underscoring the need for extended follow-up.^2^ Only a few previous studies have investigated long-term tamoxifen benefit according to luminal subtype but were limited in size and by the extent of clinical and molecular annotation. Our study includes pre- and postmenopausal patients of both high and low clinical risk, with comprehensive information on tumor characteristics and molecular subtype together with 2 decades of high-quality, complete follow-up. A key strength is the inclusion of a control group randomly assigned to no endocrine therapy.

In current clinical practice, risk stratification and treatment guidelines are commonly based on 5- to 10-year outcomes.^7^ Our studies^22^^,^^30^ as well as others^2^^,^^16^ demonstrate a substantial risk of late distant recurrence, even after tamoxifen treatment. Notably, for patients with luminal A tumors that have favorable prognosis characteristics, the early risk was low, but the separation between treated and untreated patients became more evident after 10-15 years of follow-up. This highlights the importance of extended follow-up when evaluating late recurrence risk and the benefit from tamoxifen therapy. Given the long-term benefit observed in these patients, it is clinically important whether these patients would benefit from extended tamoxifen therapy. Interestingly, a recent secondary analysis of the Investigation on the Duration of Extended Adjuvant Letrozole (IDEAL) trial suggested that patients at low genomic risk benefited from extended endocrine therapy, which was not seen for patients at higher risk.^31^

Furthermore, tamoxifen benefit was attenuated for patients with luminal B tumors with unfavorable prognosis characteristics. This attenuation in benefit was not seen in patients with luminal A subtype, and patients with luminal A larger (>20 mm) tumors had a statistically significant tamoxifen benefit, whereas for patients with large luminal B tumors, we did not observe a statistically significant benefit. Sample size was limited in some subanalyses, especially for premenopausal patients with luminal B tumors and patients with progesterone receptor–negative luminal B tumors. However, sample size was generally similar across favorable and unfavorable prognosis characteristics for patients with luminal B subtype. Because screening mammography was not yet introduced by the start of this trial, larger luminal A tumors may simply reflect longer indolent growth, whereas large luminal B tumors likely indicate more aggressive tumor biology, which then would be consistent with our overall findings on aggressivity and treatment effect. Multivariable analyses yielded similar results to crude analyses, suggesting that luminal subtype provides independent prognostic information beyond the clinically used patient and tumor characteristics.

Our results indicated that postmenopausal patients with luminal A tumors had a statistically significant benefit from tamoxifen, whereas postmenopausal patients with luminal B tumors showed a statistically significant benefit from tamoxifen in the univariate analysis but not when adjusting for patient and tumor characteristics in the multivariable analysis. These findings are consistent with our previous study showing that postmenopausal patients with favorable tumor characteristics benefited significantly from tamoxifen.^30^ Moreover, in a subset of the data consisting of low-risk postmenopausal patients (lymph node negative, pT ≤ 20 mm) we have also observed tamoxifen benefit across luminal A and B tumors, although patients with luminal B tumors experienced a larger early benefit.^6^

For premenopausal patients, we did not see a statistically significant benefit from tamoxifen for either luminal subtype, but the sample size was limited. This is especially important because premenopausal breast cancer is known to be heterogeneous,^30^ and few of the current clinically used markers adequately predict long-term tamoxifen benefit among premenopausal patients.^30^ We have previously shown that premenopausal genomic high-risk tumors may benefit more from ovarian function suppression,^22^ however the overlap between luminal B subtype and genomic high risk is only moderate.

Evolving clinical management is a limitation for all studies with long-term follow-up. Current guidelines generally recommend 20 mg of tamoxifen daily given over a longer period of time (≥5 years) compared with the 40 mg daily dose for at least 2 years given in this study. Because both dose and duration differ from today’s clinical standard, direct comparison of benefit estimates is challenging. Furthermore, the optimal duration and dose of tamoxifen are not fully understood and likely depend on multiple factors, including menopausal status. Despite the shorter duration of tamoxifen therapy used in the STO trials with the majority of patients receiving 2-year treatment, a long-term reduction of distant recurrence was observed, which can be reassuring to patients for whom extended therapy is not feasible. Additionally, the management of patients with metastatic disease has also evolved substantially over the study period, and therefore the distant recurrence-free interval was used as the primary endpoint in our study to minimize these differences. Although the overall sample size is large, caution should be taken when interpreting the results of subgroup analyses where the sample size is small.

In conclusion, our study demonstrates that tamoxifen therapy is associated with a statistically significant reduction in the long-term risk of distant recurrence in both luminal subtypes. In luminal A, the benefit is modest early but increases over time, highlighting a late benefit. In luminal B, a large early benefit is seen that persists. Subgroup analyses showed that patients with favorable tumor characteristics had a statistically significant benefit across both subtypes. The difference in timing of benefit and the substantial late risk of distant recurrence highlights the need for long-term 20-year follow-up, and shorter follow-up (<10 years) may underestimate the long-term risk and treatment benefit.

## Supplementary Material

djag049_Supplementary_Data

## Data Availability

Restrictions apply to the availability of these data according to GDPR. Data were obtained from the STO Trialist Group and are available from the authors with the permission from the STO Trialist Group.
