# Supplementary material for: Tamoxifen therapy benefit in luminal A and B breast cancer with 20-year follow-up: Secondary analysis: Stockholm Tamoxifen-2, 3, and 5 randomized controlled trials
Source: J Natl Cancer Inst. 2026 Feb 19;118(7):1248–56. doi: 10.1093/jnci/djag049 (PMC13339100; doi:10.1093/jnci/djag049)

Supplementary Material

Tamoxifen therapy benefit in luminal A and B breast cancer with 20-year follow-up

Oscar Danielsson MSc,[1,2] Huma Dar PhD,[1,2] Anna Nordenskjöld MD PhD,[3,4] Gizeh Perez-Tenorio PhD,[5] Bo Nordenskjöld MD PhD,[5] Tommy Fornander MD PhD,[1,2] Olle Stål PhD,[5] Nicholas P Tobin PhD,[1,2] Julia Tutzauer PhD,[1,2] Annelie Johansson PhD,[1,2] and Linda S Lindström PhD[1,2]

1. Department of Oncology and Pathology, Karolinska Institutet and University Hospital, Stockholm, Sweden.
2. Breast Center, Karolinska Comprehensive Cancer Center, Karolinska University Hospital, Stockholm, Sweden
3. Department of Oncology, Institute of Clinical Sciences, Sahlgrenska Academy, Gothenburg, Sweden
4. Department of Medicine, Southern Älvsborgs Hospital, Borås, Sweden
5. Department of Biomedical and Clinical Sciences and Department of Oncology, Linköping University, Linköping, Sweden.

Supplementary Methods. Details regarding randomization to endocrine therapy.

Supplementary Methods. Molecular subtyping

Table S1. Crude multivariable Cox proportional-hazards regression analysis by molecular subtype and DRFI.

Figure S1. Kaplan-Meier by luminal subtype and tamoxifen treatment.

Figure S2. Crude multivariable Cox proportional-hazards analysis of long-term tamoxifen therapy benefit by the clinically used tumor characteristics.

This supplementary material has been provided by the authors to give readers additional information about their work.

Supplementary Methods. Details regarding randomization.

All patients included in the Stockholm tamoxifen trials were clinically free of distant metastases at primary diagnosis, did not receive pre-surgery radiotherapy, neoadjuvant chemotherapy, or previous endocrine therapy, and underwent either modified radical mastectomy, or breast conserving surgery with subsequent postoperative local breast radiotherapy. Postmenopausal status was defined as not having had a menstrual cycle in the last 6 months or, if a patient had undergone hysterectomy, an age above 50 years was used. All Swedish residents have a unique personal identification number which allows automatic registry linkage to national and regional registers.[1,2] Randomization was by telephone to a central office and done using balanced lists with a random number table (permuted block technique). The allocated treatment was revealed to the clinician after randomization was registered at central office. In addition to randomization to tamoxifen therapy vs no endocrine therapy, patients in the STO-2 trial were randomly assigned to adjuvant chemotherapy versus postoperative locoregional radiotherapy. Chemotherapy was mainly by the CMF regimen (cyclophosphamide 100 mg/m2 orally day 1-14, methotrexate 40 mg/m2 intravenously on day 1 and 8, 5-fluorouracil 600 mg/m2 intravenously on day 1- 8; 12 courses over 12 months), but the first 18 months of the trial by the LMF regimen (chlorambucil 10-15 mg orally day 1-8 replaced cyclophosphamide; 12 courses up to 18 months). Locoregional radiotherapy was to the chest wall and the regional lymph nodes (46 Gy through 4.5-5 weeks). [3,4] Concurrently with endocrine therapy and as standard of care, lymph node-positive patients in STO-5 received adjuvant chemotherapy and patients with four or more positive lymph nodes also received postoperative locoregional radiotherapy. Chemotherapy was six cycles of CMF

(cyclophosphamide 600 mg/m2, methotrexate 40 mg/m2 and fluorouracil 600 mg/m2 intravenously administered days 1 to 8, every 28 days) and locoregional radiotherapy was to the chest wall, axillary- and supraclavicular lymph nodes (46 Gy through 4.5 weeks). [5,6]

Supplementary Methods. Molecular subtyping.

RNA was extracted from formalin-fixed paraffin-embedded (FFPE) tumors and analyzed using Agilent Technologies (Santa Clara, CA) gene expression microarrays. Expression intensities were linearly scaled to the 75% percentile, log transformed, and quantile normalization was applied. Gene-wise median centering was applied. Tumor molecular subtypes were defined using the PAM50 algorithm[7], which assigns each tumor to luminal A, luminal B, HER2-enriched, basal-like, or normal-like subtype by correlating expression of 50 genes to established subtype centroids. Only patients with ER-positive/HER2-negative tumors classified as luminal A or luminal B were included in our analyses.

1. Barlow L, Westergren K, Holmberg L, Talback M. The completeness of the Swedish Cancer Register: a sample survey for year 1998. *Acta Oncol*. 2009;48(1):27-33. doi:10.1080/02841860802247664

2. Brooke HL, Talbäck M, Hörnblad J, et al. The Swedish cause of death register. *Eur J Epidemiol*. Sep 2017;32(9):765-773. doi:10.1007/s10654-017-0316-1

3. Rutqvist LE, Cedermark B, Glas U, et al. The Stockholm trial on adjuvant tamoxifen in early breast cancer. Correlation between estrogen receptor level and treatment effect. *Breast Cancer Res Treat*. Dec 1987;10(3):255-66. doi:10.1007/BF01805762

4. Rutqvist LE, Johansson H. Long-term follow-up of the Stockholm randomized trials of postoperative radiation therapy versus adjuvant chemotherapy among 'high risk' pre- and postmenopausal breast cancer patients. *Acta Oncol*. 2006;45(5):517-27. doi:10.1080/02841860600702068

5. Sverrisdottir A, Johansson H, Johansson U, et al. Interaction between goserelin and tamoxifen in a prospective randomised clinical trial of adjuvant endocrine therapy in premenopausal breast cancer. *Breast Cancer Res Treat*. Aug 2011;128(3):755-63. doi:10.1007/s10549-011-1593-0

6. Sverrisdottir A, Nystedt M, Johansson H, Fornander T. Adjuvant goserelin and ovarian preservation in chemotherapy treated patients with early breast cancer: results from a randomized trial. *Breast Cancer Res Treat*. Oct 2009;117(3):561-7. doi:10.1007/s10549-009-0313-5

7. Sorlie T, Tibshirani R, Parker J, et al. Repeated observation of breast tumor subtypes in independent gene expression data sets. *Proc Natl Acad Sci U S A*. Jul 8 2003;100(14):8418-23. doi:10.1073/pnas.0932692100

Table S1. Crude multivariable Cox proportional-hazards regression analysis by molecular subtype and DRFI.

Molecular Subtype	Treatment	Patients	DRs	cHR (95% CI)[1]
Luminal A	Tamoxifen	387	83	0.60 (0.45-0.81)
	Control	301	95	1.0 (Ref.)
Luminal B	Tamoxifen	153	66	0.68 (0.48-0.96)
	Control	111	67	1.0 (Ref.)

DRFI = Distant recurrence free-interval, DRs = Distant recurrences, cHR = Crude Hazard ratio.
[1]Adjusted for age, period of primary breast cancer diagnosis, lymph node status, radiotherapy, and menopausal status, which together defined trial stratification.

Figure S1. Kaplan-Meier by luminal subtype and tamoxifen treatment. Kaplan-Meier analysis of 20-year distant recurrence-free interval (DRFI) in patients with ER-positive/HER2-negative breast cancer by luminal A and B subtype and tamoxifen therapy. Genomic risk was assessed using the 70-gene signature.

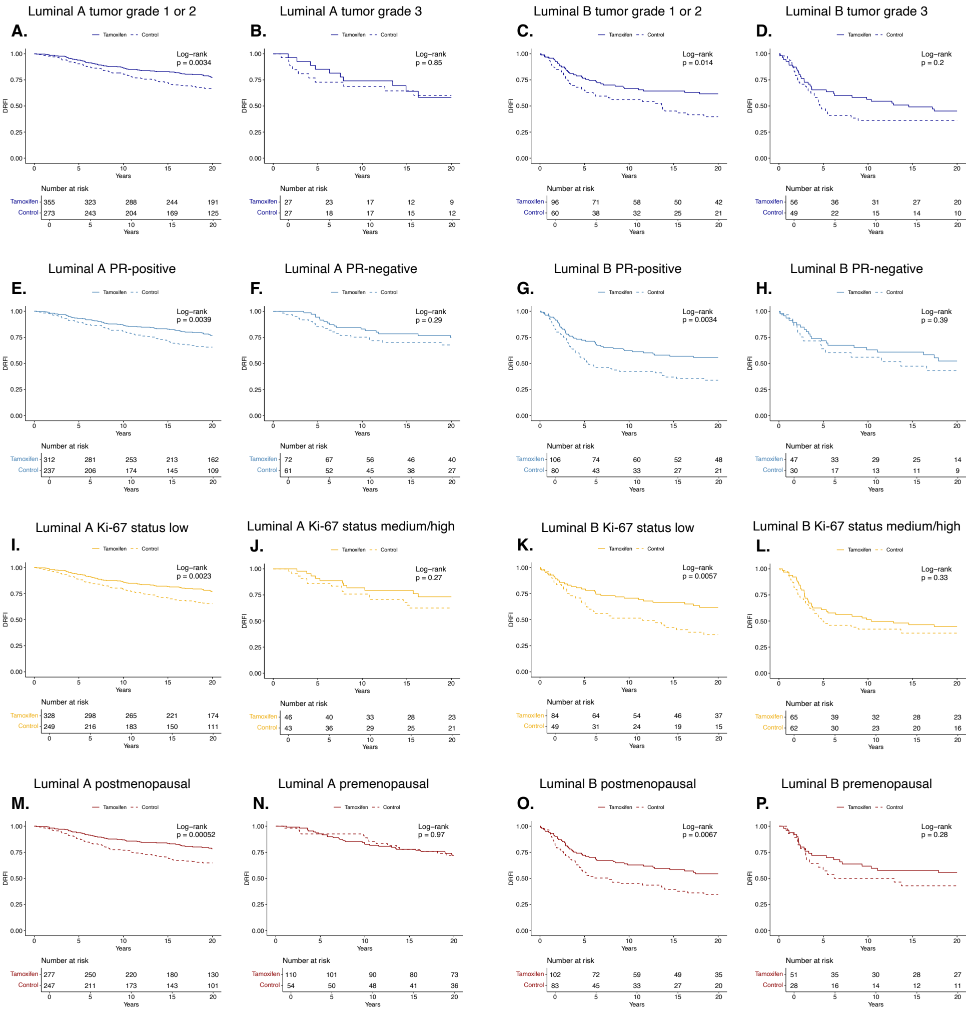

Figure S2. Multivariable Cox proportional hazards analysis of long-term tamoxifen therapy benefit by the clinically used tumor characteristics. Crude multivariable Cox proportional-hazard regression analysis of distant recurrence-free interval (DRFI) by luminal subtype in patients with ER-positive/HER2-negative breast cancer with 20 years of follow-up. Crude hazard ratio (cHR) was adjusted for age and period of primary breast cancer diagnosis, lymph node status, radiotherapy, and menopausal status, which defined trail stratification. Genomic risk was assessed using the 70-gene signature.

Luminal A

Characteristic		Trial arm	Patients No.	Distant recurrences No.	cHR (95% CI)
Tumor size	pT≤20mm	Tamoxifen	304	57	0.73 (0.50-1.06)
		Control	223	54	1.0 (Ref.)
	pT>20mm	Tamoxifen	80	26	**0.44 (0.26-0.73)***
		Control	75	41	1.0 (Ref.)
Tumor grade	1-2	Tamoxifen	355	73	**0.59 (0.43-0.81)***
		Control	273	84	1.0 (Ref.)
	3	Tamoxifen	27	10	0.97 (0.39-2.44)
		Control	27	10	1.0 (Ref.)
Lymph node status	p0	Tamoxifen	285	37	**0.50 (0.33-0.76)***
		Control	236	60	1.0 (Ref.)
	pN+	Tamoxifen	102	46	0.76 (0.49-1.19)
		Control	65	35	1.0 (Ref.)
PR status	Positive	Tamoxifen	312	65	**0.58 (0.42-0.82)***
		Control	237	75	1.0 (Ref.)
	Negative	Tamoxifen	72	17	0.61 (0.32-1.19)
		Control	61	19	1.0 (Ref.)
Ki-67 status	Low	Tamoxifen	328	69	**0.57 (0.41-0.79)***
		Control	249	80	1.0 (Ref.)
	Med/High	Tamoxifen	46	11	0.50 (0.20-1.28)
		Control	43	15	1.0 (Ref.)
Genomic Risk	Low	Tamoxifen	318	61	**0.56 (0.40-0.79)***
		Control	242	74	1.0 (Ref.)
	High	Tamoxifen	69	22	0.61 (0.32-1.16)
		Control	59	21	1.0 (Ref.)
Menopausal status	Post	Tamoxifen	277	53	**0.50 (0.35-0.71)***
		Control	247	80	1.0 (Ref.)
	Pre	Tamoxifen	110	30	0.75 (0.39-1.45)
		Control	54	15	1.0 (Ref.)

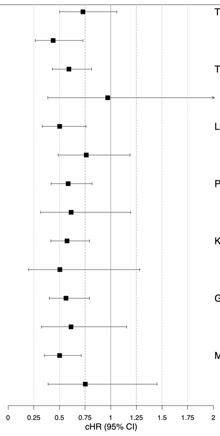

Luminal B

Characteristic		Trial arm	Patients No.	Distant recurrences No.	cHR (95% CI)
Tumor size	pT≤20mm	Tamoxifen	78	28	**0.51 (0.30-0.86)***
		Control	57	33	1.0 (Ref.)
	pT>20mm	Tamoxifen	74	37	0.88 (0.54-1.44)
		Control	53	33	1.0 (Ref.)
Tumor grade	1-2	Tamoxifen	96	35	**0.60 (0.37-0.97)***
		Control	60	35	1.0 (Ref.)
	3	Tamoxifen	56	30	0.86 (0.51-1.45)
		Control	49	30	1.0 (Ref.)
Lymph node status	p0	Tamoxifen	62	16	**0.39 (0.20-0.77)***
		Control	38	19	1.0 (Ref.)
	pN+	Tamoxifen	91	50	0.81 (0.54-1.22)
		Control	73	48	1.0 (Ref.)
PR status	Positive	Tamoxifen	106	45	**0.63 (0.42-0.95)***
		Control	80	52	1.0 (Ref.)
	Negative	Tamoxifen	47	21	0.78 (0.38-1.57)
		Control	30	15	1.0 (Ref.)
Ki-67 status	Low	Tamoxifen	84	30	**0.56 (0.33-0.96)***
		Control	49	30	1.0 (Ref.)
	Med/High	Tamoxifen	65	35	0.85 (0.53-1.36)
		Control	62	37	1.0 (Ref.)
Genomic Risk	Low	Tamoxifen	60	20	**0.44 (0.24-0.82)***
		Control	40	26	1.0 (Ref.)
	High	Tamoxifen	93	46	0.82 (0.53-1.25)
		Control	71	41	1.0 (Ref.)
Menopausal status	Post	Tamoxifen	102	44	**0.64 (0.42-0.99)***
		Control	83	51	1.0 (Ref.)
	Pre	Tamoxifen	51	22	0.60 (0.30-1.17)
		Control	28	16	1.0 (Ref.)

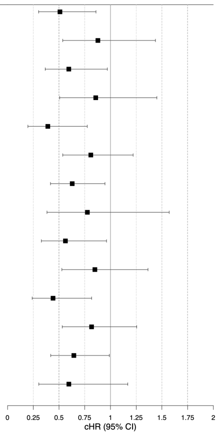

Supplement: djag049_Supplementary_Data [file djag049_supplementary_data.zip › Supplementary 13 jan 2026 JNCI.pdf]
